# Abstracts from The College of Podiatry Annual Conference 2016: Glasgow, Scotland. 17-19 November 2016

**DOI:** 10.1186/s13047-017-0191-8

**Published:** 2017-03-07

**Authors:** John Ingram, Scott Cawley, Angela Jones, Elinor Coulman, Clive Gregory, Tim Pickles, Him Shun Hinson Kei, Paul Fletcher, Mike Curran, Trevor Prior, Cynthia Formosa, Simon Otter, Keith Rome, Peter Gow, Nicola Dalbeth, Maheswaran Rohan, Sarah Stewart, Ashok Aiyer, Sam Glasser, Joanne Paton, Richard Collings, Jonathan Marsden, David Torgerson, Sarah Cockayne, Sara Rodgers, Lorraine Green, Caroline Fairhurst, Joy Adamson, Arabella Clark, Belen Corbacho, Catherine Hewitt, Kate Hicks, Robin Hull, Anne-Maree Keenan, Sarah Lamb, Hylton Menz, Anthony Redmond, Zoe Richardson, Wesley Vernon, Judith Watson, Lisa Farndon, Belen Corbacho, Sarah Cockayne, Sara Rodgers, Lorraine Green, Caroline Fairhurst, Joy Adamson, Arabella Clarke, Catherine Hewitt, Kate Hicks, Robin Hull, Anne-Maree Keenan, Sarah Lamb, Caroline McIntosh, Hylton Menz, Anthony Redmond, Zoe Richardson, Wesley Vernon, Judith Watson, Lisa Farndon, David Torgerson, Joanne Paton, Sam Glasser, Richard Collings, Jonathan Marsden, Stephen Mizzi, Lucianne Cutajar, Annabelle Mizzi, Owen Falzon, Ian Swaine, Kate Springett, Sarah Cockayne, Sara Rodgers, Lorraine Green, Caroline Fairhurst, Joy Adamson, Arabella Clarke, Belen Corbacho, Catherine Hewitt, Kate Hicks, Robin Hull, Anne-Maree Keenan, Sarah Lamb, Caroline McIntosh, Hylton Menz, Anthony Redmond, Zoe Richardson, Wesley Vernon, Judith Watson, Lisa Farndon, David Torgerson, Simon Otter, Keith Rome, Peter Gow, Nicola Dalbeth, Maheswaran Rohan, Sarah Stewart, Ashok Aiyer, Trevor Prior, Andrea Bachand, Ben Avison, Jessica Leitch, Jennifer Scott, Gordon Hendry, Jackie Locke, Carla McArdle, Katie Lagan, David McDowell, Michelle Kaminski, Anita Raspovic, Lawrence McMahon, Katrina Lambert, Bircan Erbas, Peter Mount, Peter Kerr, Karl Landorf, Louis Mamode, Catherine Bowen, Malcolm Burnett, Lucy Gates, Ann Ashburn, Mark Cole, Margaret Donovan-Hall, Ruth Pickering, Dan Bader, Judy Robison, Dorit Kunkel, Saed Al Bimani, Lucy Gates, Martin Warner, Catherine Bowen, Jane Murchie, Rachel Hannigan, Carla McArdle, Mairghread Ellis, Aimie Patience, Sophie Slater, Kirsten Wallace, Katherine Edwards, Alan M. Borthwick, Louise McCulloch, Anthony C. Redmond, Rafael Pinedo-Villanueva, Nigel K. Arden, Catherine J. Bowen, Heidi Siddle, Peter Mandl, Daniel Aletaha, Thea Vliet Vlieland, Marina Backhaus, Patricia Cornell, Maria Antonietta D’Agostino, Karen Ellegaard, Annamaria Iagnocco, Bente Jakobsen, Tiina Jasinski, Nina Kildal, Michaela Lehner, Ingrid Moller, Gabriela Supp, Philip O’Connor, Anthony Redmond, Esperanza Naredo, Richard Wakefield

**Affiliations:** 1 Department of Dermatology and Academic Wound Healing, Division of Infection & Immunity, Cardiff University, Cardiff, UK; 2 Podiatry Department, Cardiff and Vale University Health Board, Cardiff, UK; 3 Centre for Trials Research, Cardiff University, Cardiff, UK; 4 Division of Population Medicine, School of Medicine, Cardiff University, Cardiff, UK; 5 Faculty of Health and Society, University of Northampton, Northampton, UK; 6 Centre for Sports and Exercise Medicine, William Harvey Research Institute, Bart’s and the London School of Medicine and Dentistry, Queen Mary University of London, London, UK; 7 Faculty of Health Sciences, University of Malta, Msida, Malta; 8 School of Health Sciences, University of Brighton, Brighton, UK; 9 School of Podiatry, Health and Rehabilitation Research Institute, Auckland University of Technology, Auckland, New Zealand; 10 Rheumatology Department, Counties Manukau District Health Board, Auckland, New Zealand; 11 Faculty of Medical and Health Sciences, University of Auckland, Auckland, New Zealand; 12 Department of Biostatistics and Epidemiology, School of Public Health and Psychosocial Studies, Faculty of Health and Environmental Sciences, Auckland University of Technology, Auckland, New Zealand; 13 BEUP, School of Health Professions, Faculty of Health and Human Sciences, Plymouth University, Plymouth, UK; 14 Podiatry Service, Torbay and South Devon Health and Care NHS Trust, Torquay, UK; 15 York Trials Unit, Department of Health Sciences, University of York, York, UK; 16 Leeds Institute of Rheumatic and Musculoskeletal Medicine, University of Leeds, Leeds, UK; 17 NIHR Leeds Musculoskeletal Biomedical Research Unit Leeds Teaching Hospitals Trust, Leeds, UK; 18 Department of Health Sciences, University of York, York, UK; 19 Harrogate and District NHS Foundation Trust, Harrogate, UK; 20 School of Healthcare, University of Leeds, Leeds, UK; 21 Oxford NIHR Biomedical Research Unit, Oxford, UK; 22 School of Adult Health, College of Science Health and Engineering, La Trobe University, Melbourne, Australia; 23 Sheffield Teaching Hospitals NHS Foundation Trust, Sheffield, UK; 24 Jordanthorpe Health Centre, Sheffield, UK; 25 York Trials Unit, Department of Health Sciences, University of York, York, UK; 26 Leeds Institute of Rheumatic and Musculoskeletal Medicine, University of Leeds, Leeds, UK; 27 NIHR Leeds Musculoskeletal Biomedical Research Unit Leeds Teaching Hospitals Trust, Leeds, UK; 28 Department of Health Sciences, University of York, York, UK; 29 Harrogate and District NHS Foundation Trust, Harrogate, UK; 30 School of Healthcare, University of Leeds, Leeds, UK; 31 Oxford NIHR Biomedical Research Unit, Oxford, UK; 32 NUI Galway, Galway, Republic of Ireland; 33 School of Adult Health, College of Science Health and Engineering, La Trobe University, Melbourne, Australia; 34 Sheffield Teaching Hospitals NHS Foundation Trust, Sheffield, UK; 35 Jordanthorpe Health Centre, Sheffield, UK; 36 School of Health Professions, Peninsula Allied Health Centre, Plymouth University, Plymouth, UK; 37 Podiatry Service, Torbay and South Devon Health and Care NHS Trust, Torquay, UK; 38 Faculty of Health Sciences, University of Malta, Msida, Malta; 39 Faculty of Engineering, University of Malta, Msida, Malta; 40 Centre for Biomedical Cybernetics, University of Malta, Msida, Malta; 41 Department of Life and Sports Sciences, University of Greenwich, London, UK; 42 Faculty of Health and Wellbeing, Canterbury Christ Church University, Canterbury, UK; 43 York Trials Unit, Department of Health Sciences, University of York, York, UK; 44 Leeds Institute of Rheumatic and Musculoskeletal Medicine, University of Leeds, Leeds, UK; 45 NIHR Leeds Musculoskeletal Biomedical Research Unit Leeds Teaching Hospitals Trust, Leeds, UK; 46 Harrogate and District NHS Foundation Trust, Harrogate, UK; 47 School of Healthcare, University of Leeds, Leeds, UK; 48 Oxford NIHR Biomedical Research Unit, Oxford, UK; 49 NUI Galway, Galway, Republic of Ireland; 50 School of Adult Health, College of Science Health and Engineering, La Trobe University, Melbourne, Australia; 51 Sheffield Teaching Hospitals NHS Foundation Trust, Sheffield, UK; 52 Jordanthorpe Health Centre, Sheffield, UK; 53 School of Health Sciences, University of Brighton, Brighton, UK; 54 School of Podiatry, Health and Rehabilitation Research Institute, Auckland University of Technology, Auckland, New Zealand; 55 Rheumatology Department, Counties Manukau District Health Board, Auckland, New Zealand; 56 Faculty of Medical and Health Sciences, University of Auckland, Auckland, New Zealand; 57 Department of Biostatistics and Epidemiology, School of Public Health and Psychosocial Studies, Faculty of Health and Environmental Sciences, Auckland University of Technology, Auckland, New Zealand; 58 Centre for Sports and Exercise Medicine, William Harvey Research Institute, Bart’s and the London School of Medicine and Dentistry, Queen Mary University of London, London, UK; 59 Homerton University Hospital NHS Foundation Trust, London, UK; 60 Run3D Limited, The Bosworth Clinic, Oxford, UK; 61 Department for Health, University of Bath, Bath, UK; 62 School of Health and Life Sciences Department, Glasgow Caledonian University, Glasgow, UK; 63 School of Health Sciences, Queen Margaret University, Edinburgh, UK; 64 Faculty of Life and Health Sciences, Ulster University, Northern Ireland, UK; 65 Discipline of Podiatry, School of Allied Health, La Trobe University, Melbourne, Australia; 66 Department of Podiatry, Eastern Health, Melbourne, Australia; 67 Departments of Renal Medicine & Obstetric Medicine, Eastern Health Clinical School, Monash University, Melbourne, Australia; 68 College of Science, Health and Engineering, School of Psychology and Public Health, Department of Public Health, La Trobe University, Melbourne, Australia; 69 Department of Nephrology, Austin Health, Melbourne, Australia; 70 Department of Nephrology, Monash Health, Melbourne, Australia; 71 Melbourne Health, Melbourne, Australia; 72 Faculty of Health Sciences, University of Southampton, Southampton, UK; 73 Faculty of Medicine, University of Southampton, Southampton, UK; 74 Faculty of Health Sciences, University of Southampton, Southampton, UK; 75 Arthritis Research UK Centre for Sport, Exercise and Osteoarthritis, University of Southampton, Southampton, UK; 76 Arthritis Research UK Centre for Sport, Exercise and Osteoarthritis, University of Oxford, Oxford, UK; 77 Department of Physiotherapy, Institute of Health Sciences, Muscat, Oman; 78 School of Health Sciences, Queen Margaret University, Edinburgh, UK; 79 School of Health Sciences, Queen Margaret University, Edinburgh, UK; 80 Nuffield Department of Orthopaedics, Rheumatology and Musculoskeletal Sciences, University of Oxford, Oxford, UK; 81 Faculty of Health Sciences, University of Southampton, Southampton, UK; 82 Faculty of Medicine and Health, Leeds Institute of Rheumatic and Musculoskeletal Medicine, Leeds, UK; 83 MRC Lifecourse Epidemiological Unit, University of Southampton, Southampton, UK; 84 Arthritis Research Centre for Sport, Exercise and Osteoarthritis, University of Oxford, Oxford, UK; 85 Arthritis Research Centre for Sport, Exercise and Osteoarthritis, University of Southampton, Southampton, UK; 86 Leeds Institute of Rheumatic and Musculoskeletal Medicine, University of Leeds, Leeds, UK; 87 Division of Rheumatology, Medical University of Vienna, Vienna, Austria; 88 Department of Orthopaedics, Rehabilitation and Physical Therapy, Leiden University Medical Centre, Leiden, Netherlands; 89 Department of Internal Medicine, Rheumatology and Clinical Immunology, Academic Hospital of the Charité Berlin, Berlin, Germany; 90 Department of Rheumatology, Poole Hospital NHS Foundation Trust, Poole, UK; 91 >Laboratoire d’Excellence INFLAMEX, Versailles-Saint-Quentin University, Saint-Quentin-en-Yvelines, France; 92 The Parker Institute, Copenhagen University Hospital Bispebjerg-Frederiksberg, Copenhagen, Denmark; 93 Rheumatology Unit - Dipartimento di Medicina Interna e Specialità Mediche, Sapienza Università di Roma, Rome, Italy; 94 Norwegian National Advisory Unit on Pregnancy and Rheumatic Diseases, St. Olavs Hospital, Trondheim, Norway; 95 Estonian Rheumatism Association, Tallinn, Estonia; 96 Faculty of Health and Social Science, Norwegian University of Science and Technology, Trondheim, Norway; 97 Instituto Poal de Reumatologia, Barcelona, Spain; 98 Department of Radiology, Leeds Teaching Hospitals NHS Trust, Leeds, UK; 99 Department of Rheumatology, Hospital GU Gregorio Marañón, Madrid, Spain

## 1 Educational tool and point-of-care tests to assist antibiotic prescribing decisions for diabetic foot ulcer infections (INDUCE study)

### John Ingram^1^, Scott Cawley^2^, Angela Jones^2^, Elinor Coulman^3^, Clive Gregory^4^, Tim Pickles^3^

#### ^1^Department of Dermatology and Academic Wound Healing, Division of Infection & Immunity, Cardiff University, Cardiff, UK; ^2^Podiatry Department, Cardiff and Vale University Health Board, Cardiff, UK; ^3^Centre for Trials Research, Cardiff University, Cardiff, UK; ^4^Division of Population Medicine, School of Medicine, Cardiff University, Cardiff, UK

##### **Correspondence:** Angela Jones

**Background**

Assessing diabetic foot ulcers (DFUs) for infection is difficult because clinical symptoms and signs may be masked by neuropathy and vasculopathy and there are no objective tests available at point of care to guide clinicians. Empirical prescription of antibiotics compromises antibiotic stewardship, while missing early infection may lead to severe infection and amputation. In 2011-12, the cost of managing DFUs and associated amputations borne by NHS England was £650 million, nearly 1% of its budget.

Our INDUCE study had two aims: (1) to develop an online educational tool for DFU infection and (2) to conduct a pilot study investigating C-reactive protein (CRP) and procalcitonin from venous blood and calprotectin from wound exudate as inflammatory biomarkers of mild DFU infection.

**Methods**

Yola software was used to develop an online educational tool covering DFU history and examination, arterial assessment, microbiology, radiology and management of osteomyelitis. The tool contains links to NICE guidance and other relevant learning resources. A quiz using patient scenarios is included. Feedback from podiatrists was elicited by questionnaires and a focus group.

Patients with non-infected or mildly infected DFUs were recruited from community podiatry clinics in 2 UK regions. Exclusion criteria included immunosuppression or receipt of antibiotics within the previous 2 weeks. Antibiotics were prescribed based on clinical judgement at the baseline assessment. Our gold standard defining DFU infection was the clinician’s judgement one week later, while still blinded to test results, factoring in the response to antibiotic therapy, if prescribed. All 3 inflammatory biomarkers were measured at weeks 0 and 1, including assessment of CRP using a point-of-care device.

**Results**

Feedback regarding the educational tool from end-users ranging from trainee to senior podiatrists was very positive. The main improvement requested was a printable certificate after successful completion of the quiz and provision of CPD points.

Between September 2014 and September 2015, the INDUCE study recruited 67 patients with DFUs, from a total of 363 potential participants. Primary endpoints were available for 37 participants with non-infected ulcers and 28 with mild infection, following early study withdrawal by one patient in each group. Median CRP was slightly higher in the infected ulcer group, 7.50 mg/ml compared to 6.00 mg/ml for non-infected ulcers, but the area under the receiver operating characteristic curve (AUROC) was only 0.52, demonstrating poor predictive efficacy. Most of the procalcitonin results were below the lower limit of the assay and levels were lower in the infected DFU group. Median calprotectin levels were nearly doubled in infected ulcers, 1437 ng/ml compared with 879 ng/ml in non-infected DFUs, but with an insufficient AUROC of 0.56.

**Conclusions**

Feedback from a range of podiatrists confirmed that assessment of DFU infection remains challenging and showed that the INDUCE tool is a useful learning resource. The tool will be made freely available via the internet.

Based on their sensitivity and specificity, neither venous CRP or procalcitonin should be pursued as biomarkers of DFU infection, alone or in combination. Calprotectin in wound exudate may have value, but only in combination with other biomarkers.

## 2 Shoe hardness and gait

### Him Shun Hinson Kei, Paul Fletcher, Mike Curran

#### Faculty of Health and Society, University of Northampton, Northampton, UK

##### **Correspondence:** Him Shun Hinson Kei

**Introduction**

This study will have implications for any healthcare professionals who aim to alter patient’s gait with footwear. This study examines the reliability of Dartfish 8 with Canon 700D in the measurement of joint motion and percentages of different stance phases. It also serves as a pilot study to investigate the influence of sole hardness on the walking gait to recommend footwear and prevent injury.

**Methods**

14 participants walked along a walkway at their preferred speed under four conditions of different sole hardness (barefoot, 58 shore C, 68 shore C and 74 shore C). The sequence of the shoe conditions was randomly assigned. Ankle joint and first metatarsophalangeal joint (1st MTPJ) maximum dorsiflexion and the percentage of different stance phases were determined with Dartfish 8.0. Intra-class Correlation Coefficient Test was carried out to check if the two measures for each angle concerned and each percentage concerned had absolute agreement using the two-way mixed model. Statistical techniques were used to identify differences among different sole conditions.

**Results**

The presented protocol was reliable in the measurement of ankle and 1st MTPJ maximum dorsiflexion but not as accurate in the measurement of the percentages of different stance phases. Left ankle maximum dorsiflexion was greater with moderate soles compared to hard soles and with soft soles compared to barefoot. The Differences observed in 1st MTPJ maximum dorsiflexion were minimal. The percentage of midstance for the left foot was larger in the barefoot condition than the soft and moderate sole conditions. Conclusion: For the 14 participants in this pilot study, moderate soles encourage left ankle range of motion (ROM) whereas hard soles restrict left ankle ROM. A limited range of motion has been cited as a cause of injury and a risk factor for diabetic ulceration. This pilot study may be particularly important in the management of diabetic patients and patients who walk for a long time routinely. The effect of sole hardness on joint motion and gait phases still warrant further investigation. It may be worth examining the long-term effect of sole hardness on different joints and the walking gait. It is hoped that optimal sole hardness could be recommended to patients based on their age, weight and biomechanical presentation in the future.

## 3 Plantar loading patterns of elite rowers on a fixed ergometer: Comparison of a commercially available rowing specific shoe to a training shoe

### Trevor Prior

#### Centre for Sports and Exercise Medicine, William Harvey Research Institute, Bart’s and the London School of Medicine and Dentistry, Queen Mary University of London, London, UK

**Background**

In the sport of rowing, load applied through the feet must be efficiently and effectively translated into propulsive force at the oar. Factors inherent to this process include; technique, anthropometric variables and equipment. By studying forces applied at the foot, one can develop a model of the most effective loading pattern. By comparing these loading patterns between equipment variables, such as footwear, an ideal setup can be chosen based on the athletes’ specific anthropometry or technique. It could even be possible to advise the athlete, based on the observed loading pattern, how to adjust technique to achieve greater performance. The first aim was to build on previous research regarding plantar load in rowing using an inshoe plantar pressure device. The second, to compare loading patterns using a commercially available rowing shoe, to a training shoe.

**Methods**

10 elite male university rowers conducted two identical rowing trials, once with a training shoe (TS) and once with a commercially specific rowing shoe (RS). The ergometer was instrumented at the stretcher with a capacitive insole placed in each shoe. The foot was masked, subdividing into 7 anatomical areas; heel, midfoot, 1st Metatarsal head, 2nd + 3rd Metatarsal heads, 4th +5th Metatarsal heads, 1st digit and 2nd -5th digits. For each area and the total surface outcomes recorded include: Maximum force (N), Instant of maximum force (%ROP), Force-time integral (N*s), Mean pressure (kPa), Peak pressure (kPa), Instant of peak pressure (%ROP), Pressure-time integral (kPa*s), Contact area (cm2), Begin of contact (%ROP), End of contact (%ROP), Contact time (p) (%ROP).

**Results**

Maximum force is greater over the 1st Met head in RS (p < 0.01). Instant of maximum force occurred earlier over the 2nd-3rd Met head in RS (p = 0.027). Force time integral is greater over the 1st Met head in RS (p < 0.01). Peak pressure and pressure time integral is higher over total area and 1st Met head in RS (p < 0.01 for all). Contact area is greater in TS over all areas but 3rd-5th digits (p < 0.01 for all). Of the anatomical areas; Maximum force is highest over the heel in TS and 1st Met head in RS, 2nd-3rd Met had the greatest Force time integral in RS and TS, highest peak pressures under the 1st Met head in RS and TS and highest pressure time integral under the 1st Met head in RS and TS.

**Conclusions**

The softer nature and size of the training shoe allow for a greater spread of the force beneath the foot during the rowing stroke and facilitated heel contact. By contrast, the rowing shoe focussed the force beneath the 1st Met head and, due to the reduced contact area, resulted in a greater peak pressures.

## 4 Importance of accurate identification of arterial perfusion using different physiological tests

### Cynthia Formosa

#### Faculty of Health Sciences, University of Malta, Msida, Malta

**Background**

Although diagnostic and therapeutic decisions in patients with peripheral arterial disease are guided primarily by the history and physical examination, the use of non-invasive investigations has increased significantly in recent years, mainly as a result of technological advances in ultrasonography. Ultrasonic hand held doppler velocimeters are widely utilized by healthcare professionals for the assessment of lower limb arterial perfusion. However, a clinical assessment tool is only of value if the interpretation of the results is correct and repeatability has been clinically established. Ankle Brachial Pressure Index, TBIs, toe pressures and spectral waveforms at the ankle are all used to assess arterial perfusion especially in high risk populations.

**Design**

Single centre prospective non-experimental study design.

**Methods**

This paper presents the findings of two studies conducted to compare the accuracy of different physiological tests used by various health care professionals for the identification of arterial perfusion in the foot. Vascular testing included assessment of arterial spectral waveforms at the ankle, absolute toe pressures, toe-brachial pressure index (TBI), ankle-brachial pressure index (ABPI) and pulsatiliy index (PI).

**Results**

TBIs, toe pressures and spectral waveforms at the ankle are better predictors of likelihood of healing and non-healing after minor amputation than ABPIs. ABPI alone is a poor indicator of the likelihood of healing of minor amputations and should not be relied on to determine the need for revascularization procedures in the high risk foot. The PI, being a continuous variable allows classification of patients on a scale, making it easier to diagnose severity of disease, thus resulting in a more accurate interpretation of the extent of disease. The advantage of PI over spectral waveforms is that it is a quantitative measure, rather than a subjective assessment of spectral waveforms thus ensuring no variability in user interpretation between waveforms especially in severely diseased arteries which can prove to be difficult to interpret correctly.

**Conclusions**

Results from the two studies presented support the evidence that toe pressures above 30 mmHg could predict healing as proposed by the European Consensus Document [ECD] but also indicate that spectral waveforms at the ankle are also good predictors of arterial perfusion. As importantly it highlights the fact that reliance on ABPIs to predict accurate perfusion is inadvisable. Results also conclude that pulsatility index can safely be used to identify patients’ with peripheral arterial disease. Using PI, which is a quantitative measure, rather than a subjective assessment of spectral waveforms removes the variability of user interpretation.

## 5 Foot and ankle muscle strength in people with gout and/or diabetes

### Simon Otter^1^, Keith Rome^2^, Peter Gow^3^, Nicola Dalbeth^4^, Maheswaran Rohan^5^, Sarah Stewart^2^, Ashok Aiyer^2^

#### ^1^School of Health Sciences, University of Brighton, Brighton, UK; ^2^School of Podiatry, Health and Rehabilitation Research Institute, Auckland University of Technology, Auckland, New Zealand; ^3^Rheumatology Department, Counties Manukau District Health Board, Auckland, New Zealand; ^4^Faculty of Medical and Health Sciences, University of Auckland, Auckland, New Zealand; ^5^Department of Biostatistics and Epidemiology, School of Public Health and Psychosocial Studies, Faculty of Health and Environmental Sciences, Auckland University of Technology, Auckland, New Zealand

##### **Correspondence:** Simon Otter

**Background**

Gout is the most common form of inflammatory arthritis in men and diabetes is increasing in prevalence across the World. Gout and diabetes are associated with major functional and structural lower limb and significant foot impairments, including mobility loss, foot ulceration and impaired gait. We aimed to determine the differences in foot and ankle muscle strength between people with gout, diabetes, both gout and controls.

**Methods**

In total, 112 participants were recruited, (35 gout, 28 diabetes, 20 gout and diabetes, 29 controls) were recruited from a rheumatology and podiatry departments in Auckland, New Zealand. Gout participants met the 1977 American Rheumatism Association classification criteria for gout and diabetes confirmed through hospital records (Trial registration: ACTRN12614001140640).

Age, gender, ethnicity, body mass index (BMI), current medications and co-morbidities were recorded. Gout disease duration, serum urate and presence of subcutaneous tophi were recorded for gout participants. HbA1C was recorded for diabetes participants. Additionally, serum creatinine and estimated glomerular filtration rate were recorded.

General body pain and patient global were assessed using 100 mm Visual Analog Scales (VAS). The Health Assessment Questionnaire - Disability Index (HAQ-DI) was used to measure activity limitation. Patient reported foot pain and disability was assessed using the Manchester Foot Pain and Disability Index (MFPDI). Sensory testing, via vibration perception threshold was assessed using a biothesiometer. Isometric muscle force for foot and ankle plantarflexion, dorsiflexion, inversion and eversion was measured using hand-held dynamometry.

One-way ANOVAs (for continuous data) and Kruskal-Wallis tests (for categorical data) were used to determine differences between groups. Significant differences between groups for each muscle test condition were assessed with mixed linear models. Models were adjusted for age, sex and BMI. Models accounted for repeated measures taken from right and left feet of each participant through adopting a mixed models approach in which a participant-specific random effect and participant-nested random effect for foot-side were included.

**Results**

Participants with gout and/or diabetes had greater BMI, more frequent hypertension, hyperlipidaemia and chronic kidney disease, compared to control participants (p < 0.001). After adjusting for age, sex and BMI, participants with gout, diabetes and both gout and diabetes had reduced plantarflexion dorsiflexion and inversion strength compared to controls (all p < 0.001). Additionally, participants with diabetes had reduced plantarflexion strength compared to participants with gout (p = 0.045). Participants with gout (p = 0.003), diabetes (p < 0.001), and both conditions (p = 0.001) also demonstrated reduced eversion strength compared to the control group.

**Conclusions**

Patients with gout, diabetes and co-existing gout and diabetes have impaired muscle strength in foot and ankle plantarflexion, dorsiflexion, eversion and inversion when compared to individuals with neither condition. These data highlight the importance of foot and ankle strengthening exercises in individuals with chronic foot conditions to prevent or reduce lower limb disability and the need for future research assessing the benefits of foot and ankle strengthening exercises to improve lower limb function in people with chronic comorbid conditions.

## 6 The effects of foot and ankle orthoses and stance width in people with diabetic peripheral neuropathy

### Sam Glasser^1,2^, Joanne Paton^1^, Richard Collings^1,2^, Jonathan Marsden^1^

#### ^1^BEUP, School of Health Professions, Faculty of Health and Human Sciences, Plymouth University, Plymouth, UK; ^2^Podiatry Service, Torbay and South Devon Health and Care NHS Trust, Torquay, UK

##### **Correspondence:** Sam Glasser

**Background**

Falls are a major concern for people with diabetic peripheral neuropathy (DPN). Ankle foot orthoses (AFO) may provide a cost-effective approach to enhancing balance and reducing falls risk by improving ankle stability. Most of the research on the topic is focused on AFO’s designed to limit ankle joint dorsiflexion. Bracing medio-lateral foot-ankle movement in people with DPN has been largely overlooked. It is well documented that as a complication of diabetes, the reduction in the elasticity of the structures surrounding the ankle reduces ankle range of motion. This altered biomechanics has been associated with increased forefoot pressures and increased ulceration risk. The use of an AFO designed to reduce ankle dorsiflexion movement further may have a similar negative effect on peak pressure. An alternative approach could be to use an AFO designed to only restrict medio-lateral rear foot motion as a clinically safe intervention to enhance balance without increasing plantar pressures for people with diabetes and neuropathy.

**Aim**

The aim of this study was to explore the effect of AFO and stance width on standing balance in people with DPN compared to a healthy control group.

**Methods**

18 subjects per group were investigated (DPN vs Healthy). Participants stood in standardised footwear (Pullman, USA) with their eyes closed. The medial borders of their feet were either 4 or 16 cm apart. For each stance width people stood in 2 conditions; “AFO” or “No AFO”. The order of the stance width and AFO use was randomised. After a random (1-3 seconds) period frontal plane movement of markers placed on the shoulders (acromion process) was recorded during a 1 second baseline period using 3D motion analysis (CodaMotion, UK). The centre point between shoulder markers was calculated in MatLab (Mathworks UK) for subsequent calculation of sway velocity.

**Results**

The effects of stance width and AFO on the response to hip vibration was compared using a between groups repeated measures ANOVA. Sway velocity was measured and analysed across both groups for effects of Group, AFO, Stance width and their interactions. There was an effect of group (P < 0.05) with the diabetic group having a higher baseline sway. There was also an effect of AFO, with the AFO reducing sway (P < 0.001) across both groups. An increase in stance width was associated with a reduction in sway in both groups (P = 0.00)). A Stance width*AFO (P = 0.04) interaction highlighted that the effect of the AFO on reducing sway velocity was more marked at 4 cm stance width compared to 16 cm stance width.

**Discussion**

To our knowledge this is the first study to highlight the potential beneficial effects of medio-lateral ankle brace in those with DPN. By stabilising medio-lateral ankle/foot motion with AFO’s, increased stability was indicated by a reduction in sway velocity. This study has provided further exploratory findings about the effects of AFO’s and stance width on sway velocity. It is intended that the findings will be used to inform the development of an AFO suitable for clinical use in a clinical trial targeted at the neuropathic population.

## 7 Clinical effectiveness of a multifaceted podiatry intervention for falls prevention in older people: A multicentre cohort Randomised Controlled Trial (REFORM)

### David Torgerson^1^, Sarah Cockayne^1^, Sara Rodgers^1^, Lorraine Green^2,3^, Caroline Fairhurst^1^, Joy Adamson^4^, Arabella Clark^1^, Belen Corbacho^1^, Catherine Hewitt^1^, Kate Hicks^1^, Robin Hull^5^, Anne-Maree Keenan^3,6^, Sarah Lamb^7^, Hylton Menz^8^, Anthony Redmond^2,3^, Zoe Richardson^1^, Wesley Vernon^9^, Judith Watson^1^, Lisa Farndon^10^

#### ^1^York Trials Unit, Department of Health Sciences, University of York, York, UK; ^2^Leeds Institute of Rheumatic and Musculoskeletal Medicine, University of Leeds, Leeds, UK; ^3^NIHR Leeds Musculoskeletal Biomedical Research Unit Leeds Teaching Hospitals Trust, Leeds, UK; ^4^Department of Health Sciences, University of York, York, UK; ^5^Harrogate and District NHS Foundation Trust, Harrogate, UK; ^6^School of Healthcare, University of Leeds, Leeds, UK; ^7^Oxford NIHR Biomedical Research Unit, Oxford, UK; ^8^School of Adult Health, College of Science Health and Engineering, La Trobe University, Melbourne, Australia; ^9^Sheffield Teaching Hospitals NHS Foundation Trust, Sheffield, UK; ^10^Jordanthorpe Health Centre, Sheffield, UK

##### **Correspondence:** Caroline Fairhurst

**Background**

Approximately a third of adults aged over 65 years fall each year. Falls and related injuries are a serious cause of morbidity and cost to individuals and society. Evidence suggests that foot problems and inappropriate footwear can cause falls. Podiatric interventions could potentially help reduce falls; however, there is limited evidence as to their clinical and cost-effectiveness.

**Methods**

The objective of this pragmatic, multi-centred, cohort Randomised Controlled Trial was to determine the clinical and cost-effectiveness of a multifaceted podiatry intervention in preventing falls in community dwelling older people, relative to usual care. An economic evaluation and qualitative study were embedded. Men and women aged 65 years and over with an increased risk of falling were recruited from nine NHS trusts based in primary and secondary care in the UK and one international site in the Republic of Ireland. Participants were randomised either to the Intervention group (n = 493) or to Usual Care (n = 517), with 12 months’ follow-up. Treatment allocations were computer-generated and conducted via a secure, remote randomisation service. Groups of participants from a centre were randomised in a single block mostly in a 1:1 ratio; however, unequal randomisation was used if the clinic had capacity to schedule appointments for more or less than half the group size. Neither participants nor podiatrists were blinded to group allocation. The intervention consisted of: footwear advice, footwear provision if required, foot orthoses, foot and ankle strengthening exercises, and a falls prevention leaflet. Participants in the Usual Care group received the falls prevention leaflet in addition to routine care from their podiatrist and GP. The primary outcome measure was the number of falls per participant in the 12 months following randomisation. Secondary outcome measures included: proportion of fallers and those reporting multiple falls, time to first fall, fear of falling, Short Falls Efficacy Scale – International, Frenchay Activities Index, Geriatric Depression Scale, two-item Connor-Davidson Resilience Scale, foot pain, fracture rate, and cost-effectiveness.

**Results**

The primary analysis included 991 participants (484 (98.2%) in the Intervention group and 507 (98.1%) in Usual Care). There was a non-statistically significant reduction in the incidence rate of falls in the Intervention group relative to Usual Care (adjusted incidence rate ratio 0.88, 95% CI 0.73 to 1.05, p = 0.16). The proportion of participants experiencing a fall, or multiple falls, was significantly lower in the Intervention group (50% vs 55%, adjusted odds ratio (OR) 0.78, 95% CI 0.60 to 1.00, p = 0.05; and 28% vs 35%; adjusted OR 0.69, 95% CI 0.52 to 0.90, p = 0.01, respectively). No differences were observed in any of the other secondary outcomes. No serious, unexpected and related adverse events were reported.

**Conclusions**

The multifaceted package of podiatry care was seen to be a safe and relatively effective intervention in reducing the proportion of older adults who experience a fall over 12 months. Although the primary outcome measure (incidence rate of falls) did not reach significance, a benefit was observed in the Intervention group. The results of the economic evaluation suggest that the intervention could be a cost-effective option for falls prevention.

## 8 Cost-effectiveness analysis of the REFORM trial: a randomised controlled trial comparing a multifaceted podiatry intervention versus usual care for falls prevention

### Belen Corbacho^1^, Sarah Cockayne^1^, Sara Rodgers^1^, Lorraine Green^2,3^, Caroline Fairhurst^1^, Joy Adamson^4^, Arabella Clarke^1^, Catherine Hewitt^1^, Kate Hicks^1^, Robin Hull^5^, Anne-Maree Keenan^3,6^, Sarah Lamb^7^, Caroline McIntosh^8^, Hylton Menz^9^, Anthony Redmond^2,3^, Zoe Richardson^1^, Wesley Vernon^10^, Judith Watson^1^, Lisa Farndon^11^, David Torgerson^1,11^

#### ^1^York Trials Unit, Department of Health Sciences, University of York, York, UK; ^2^Leeds Institute of Rheumatic and Musculoskeletal Medicine, University of Leeds, Leeds, UK; ^3^NIHR Leeds Musculoskeletal Biomedical Research Unit Leeds Teaching Hospitals Trust, Leeds, UK; ^4^Department of Health Sciences, University of York, York, UK; ^5^Harrogate and District NHS Foundation Trust, Harrogate, UK; ^6^School of Healthcare, University of Leeds, Leeds, UK; ^7^Oxford NIHR Biomedical Research Unit, Oxford, UK; ^8^NUI Galway, Galway, Republic of Ireland; ^9^School of Adult Health, College of Science Health and Engineering, La Trobe University, Melbourne, Australia; ^10^Sheffield Teaching Hospitals NHS Foundation Trust, Sheffield, UK; ^11^Jordanthorpe Health Centre, Sheffield, UK

##### **Correspondence:** Belen Corbacho

**Background**

A two arm, pragmatic, open, multicentre randomised controlled trial (REFORM) was conducted in nine National Health Service (NHS) trusts based in primary and secondary care in the UK and one international site in the Republic of Ireland to evaluate the clinical effectiveness and cost-effectiveness of a multifaceted podiatry intervention compared to usual care for the prevention of falls.

**Methods**

Individual patient data were used to perform a cost utility analysis where health related quality of life (HRQoL) was measured in terms of quality adjusted life years (QALYs). Health care resource use data included hospital visits and primary and community care. The base case analysis was conducted on the imputed dataset following an intention-to-treat (ITT) approach and from the perspective of the UK NHS and personal social services. Costs were expressed in £ GBP at a 2015 price base. Differences between the intervention and usual care treatment groups in costs and QALYs at one year were used to derive an estimate of the cost-effectiveness of the multifaceted intervention using regression methods.

**Results**

The base case analysis shows that the participants randomised to the podiatry intervention experienced (marginally) better health outcomes. At the end of the trial, the intervention group had experienced 0.0129 (95% CI: -0.0050; 0.0314) more QALYs but is also more expensive, as the podiatry intervention costs on average £252.17 more per participant when compared with usual care (95% CI: -69.48 to 589.38) when adjusted for baseline covariates. ICERs range between £19,494 and £20,593 per additional QALY. According to NICE, the willingness to pay (WTP) threshold for an additional QALY ranges from £20,000 to £30,000. The probability of the podiatry intervention being the more cost-effective option is above 0.60 both for the base-case and secondary analysis (societal perspective) at a willingness to pay threshold of £30,000 per QALY. The results are robust to the scenario analyses testing assumptions regarding resource use, perspective of analysis and level of imputation regarding missing data on HRQoL.

**Conclusions**

The results of the economic evaluation conducted alongside the REFORM trial suggest that the multifaceted intervention is a cost-effective option for falls prevention. However, the short follow-up of the study might be insufficient to take into account for differences in costs and QALYs that may be expected over the longer term. If we make the assumption that falls reduction should also lead to a fracture reduction, it is likely that the podiatric intervention might yield to long term cost savings within the NHS. We therefore recommend a longer-term model to explore whether the multifaceted intervention would become more cost-effective over time.

## 9 Does the severity of diabetic peripheral neuropathy alter the effect of insoles on balance?

### Joanne Paton^1^, Sam Glasser^1^, Richard Collings^2^, Jonathan Marsden^1^

#### ^1^School of Health Professions, Peninsula Allied Health Centre, Plymouth University, Plymouth, UK; ^2^Podiatry Service, Torbay and South Devon Health and Care NHS Trust, Torquay, UK

##### **Correspondence:** Joanne Paton

**Background**

People with Diabetic Peripheral Neuropathy (DPN) routinely wear offloading insoles to reduce foot ulcer risk. It is implicated that such insoles impede balance by dampening any remaining sensory input. Likewise, the usefulness of textured insoles designed to optimise residual sensory awareness are considered useless in those completely unable detect texture. However there has been little research to determine if the effect of insoles on postural balance differs with DPN severity. It is clinically important to understand how the potential benefits and risks of insole therapy differ within the intended clinical populations.

**Objectives**

To compare differences in the effect of a standard offloading insole and its constituent parts on postural balance in people with moderate and severe DPN.

**Methods**

In this sub group analysis, 21 people with severe DPN, and 27 people with moderate DPN, were observed standing for three 30 second trials, with eyes closed, under five test conditions presented in a random order; 1) no insole, 2) standard offloading insole, 3) three other insole types with one design component systematically altered (including texture). The validated F-scan pressure measurement system captured movement of the centre of pressure (COP). Total velocity of COP and velocity of COP in the medial/lateral (ML), anterior/posterior (AP) directions were calculated. Likewise, total path length of COP and path length in the ML and AP directions were calculated. DPN severity was quantified using a neurothesiometer applied to the apex of the hallux. Mixed between-within subject ANOVA, using data collected under each test condition were used to compare differences in postural balance between moderate (vibration perception threshold VPT range: 20-40 volts, mean age: 72 years) and severe (VPT: >40 volts; mean age 68 years) DPN groups.

**Results**

No significant interaction between DPN severity and insole condition, p > 0.05 was found. Comparison of moderate and severe DPN findings were not significant for any of the postural sway parameters (p > 0.05), suggesting that DPN severity makes no difference to the effect of the insoles. There was a significant main effect between insole test conditions for all measures of postural sway (p0.05).

**Conclusions**

The effect of insoles on postural balance is independent of neuropathy severity. The standard offloading insole increased postural sway in people with DPN but this effect was countered by; 1. the addition of a textured cover, 2. The removal of the arch fill. People with DPN, who display poor postural balance when wearing the standard offloading insole, may benefit from wearing a flat insole. Textured insoles appear to alter sensory awareness even in those with severe neuropathy. The effect of texture on balance, gait and falls in all people with DPN (regardless of neuropathy severity) merits further investigation.

## 10 Influence of ambient temperature on in-shoe foot temperature kinetics during a 40-minute treadmill walk

### Stephen Mizzi^1^, Lucianne Cutajar^2^, Annabelle Mizzi^1^, Owen Falzon^3^, Ian Swaine^4^, Kate Springett^5^

#### ^1^Faculty of Health Sciences, University of Malta, Msida, Malta; ^2^Faculty of Engineering, University of Malta, Msida, Malta; ^3^Centre for Biomedical Cybernetics, University of Malta, Msida, Malta; ^4^Department of Life and Sports Sciences, University of Greenwich, London, UK; ^5^Faculty of Health and Wellbeing, Canterbury Christ Church University, Canterbury, UK

##### **Correspondence:** Stephen Mizzi

**Background**

There are two competing drivers for foot temperature during exercise and they seem to be related to thermoregulatory vasodilation and reflex vasoconstriction, associated with the exercise. However, the pattern of change in foot temperature kinetics during exercise has not been described previously. This study provides continuous measurement data of ambulatory foot temperature in different ambient temperatures aiming to provide insight into the influence of climate on ambulatory foot temperature kinetics. These results, in healthy participants provide the first data on foot temperature kinetics during exercise in individuals living in a Mediterranean climate, and provide the foundation for the use of this type of technology in clinical contexts including the evaluation of patients at risk of foot ulceration.

**Methods**

Fourteen healthy individuals (5 males, 9 females) were recruited to assess the influence of ambient temperature on in-shoe micro climate during ambulation by comparing foot temperature kinetics during Mediterranean winter and summer seasons. A dedicated thermistor was placed on two locations of the foot – the web space between the hallux and second toe and below the navicular. After acclimatizing for 15 minutes, participants walked on a treadmill for 40 minutes while continuously recording foot temperature. This protocol was repeated on two different occasions representing ambient winter (17.1 °C at 67% RH) and summer (28.2 °C at 70% RH) conditions, at the same treadmill speed and whilst wearing the same socks and shoes, on both occasions. Data was recorded every minute throughout the 40-minute trial.

**Results**

Foot temperature kinetics approximated a sigmoidal shaped curve in both seasons, displaying a much ‘flatter’ s-shape during summer. Overall, ambient temperature had a significant influence on in-shoe forefoot and midfoot temperatures during the 40 minutes of moderate physical exercise (Paired Sample T-test; p = < 0.05). Results demonstrated that in an ambient temperature of 28.2 °C (±0.6) typical of summer season in a Mediterranean climate, foot temperature during exercise increased by 3 °C (from 33 °C to 36 °C). A different foot temperature kinetic pattern was evident in winter with temperature increasing by 7 °C (from 27 °C to 34 °C) over 40-minutes of exercise when the ambient temperature was 17.1 °C (±0.4).

**Conclusion**

This study has shown a distinct difference between summer and winter in in-shoe temperature kinetics demonstrating that ambient temperature has a significant influence on foot temperature kinetics during exercise in healthy participants, revealing that thermoregulatory function may be reflected in these measurements. To date there has been relatively little study of in-shoe foot temperature kinetics during physical exercise in relation to ambient temperature. Therefore, this study provides baseline data for comparison with diabetic participants where any difference detected may reveal presence of thermoregulatory dysfunction and impaired microcirculation with implications to diabetic foot ulceration development.

## 11 The REFORM trial – views and experiences of podiatrists on the delivery of a multifaceted intervention for the prevention of falls in older people

### Sarah Cockayne^1^, Sara Rodgers^1^, Lorraine Green^2,3^, Caroline Fairhurst^1^, Joy Adamson^1^, Arabella Clarke^1^, Belen Corbacho^1^, Catherine Hewitt^1^, Kate Hicks^1^, Robin Hull^4^, Anne-Maree Keenan^3,5^, Sarah Lamb^6^, Caroline McIntosh^7^, Hylton Menz^8^, Anthony Redmond^2,3^, Zoe Richardson^1^, Wesley Vernon^9^, Judith Watson^1^, Lisa Farndon^10^, David Torgerson^1^

#### ^1^York Trials Unit, Department of Health Sciences, University of York, York, UK; ^2^Leeds Institute of Rheumatic and Musculoskeletal Medicine, University of Leeds, Leeds, UK; ^3^NIHR Leeds Musculoskeletal Biomedical Research Unit Leeds Teaching Hospitals Trust, Leeds, UK; ^4^Harrogate and District NHS Foundation Trust, Harrogate, UK; ^5^School of Healthcare, University of Leeds, Leeds, UK; ^6^Oxford NIHR Biomedical Research Unit, Oxford, UK; ^7^NUI Galway, Galway, Republic of Ireland; ^8^School of Adult Health, College of Science Health and Engineering, La Trobe University, Melbourne, Australia; ^9^Sheffield Teaching Hospitals NHS Foundation Trust, Sheffield, UK; ^10^Jordanthorpe Health Centre, Sheffield, UK

##### **Correspondence:** Arabella Clarke

**Background**

The REFORM trial evaluated a multifaceted podiatry intervention for the prevention of falls in older people consisting of: footwear advice and provision if required, orthosis and a program of foot and ankle exercises. This qualitative study was undertaken to examine the views and experiences of the podiatrists who had delivered the REFORM intervention.

**Methods**

Semi-structured interviews were conducted face-to-face or over the telephone with 15 podiatrists who had delivered the trial intervention. Podiatrists represented seven NHS trusts in the UK and a University Podiatry School in the Republic of Ireland. Interviews lasted between 30 and 70 minutes. Following transcription, interviews were analysed thematically - data codes and themes were refined through discussion with an independent researcher.

**Results**

The findings from the qualitative interviews with podiatrists are reported according to the three components of the REFORM trial intervention; footwear assessment and provision, the orthosis and the exercises.

Footwear: Podiatrists assessed, advised about and, if required, provided footwear. Footwear assessment and advice is provided in routine podiatry care but not strictly in relation to falls prevention. Podiatrists acknowledged the importance of providing advice for falls prevention but had concerns about implementing this in routine podiatry care in terms of the impact on consultation time. Footwear provision is not part of routine podiatry care (unless in cases of severe deformity) but was described as an important facilitator for patients changing their footwear.

Orthosis: Podiatrists reported a preference for the trial ‘x-line’ orthosis over other routinely prescribed orthoses and described it as good, cost-effective and easy to fit in patients’ shoes.

Exercises: In contrast to routine care, where specific exercises are prescribed for specific lower limb pathologies, a general package of foot and ankle exercises was prescribed for the trial. Despite largely positive experiences of the exercise package, practical issues were reported including: prescribing/adapting exercises for elderly and frail patients and the number of exercises that were prescribed.

**Conclusions**

Insights into the potential to include interventions of this type in routine podiatry practice will be discussed including: appointment length, professional boundaries and adapting exercises to an older and potentially frail population.

Trial registration ISRCTN68240461 Date assigned 01/07/2011

## 12 Foot function and activity limitation in people with gout

### Simon Otter^1^, Keith Rome^2^, Peter Gow^3^, Nicola Dalbeth^4^, Maheswaran Rohan^5^, Sarah Stewart^2^, Ashok Aiyer^2^

#### ^1^School of Health Sciences, University of Brighton, Brighton, UK; ^2^School of Podiatry, Health and Rehabilitation Research Institute, Auckland University of Technology, Auckland, New Zealand; ^3^Rheumatology Department, Counties Manukau District Health Board, Auckland, New Zealand; ^4^Faculty of Medical and Health Sciences, University of Auckland, Auckland, New Zealand; ^5^Department of Biostatistics and Epidemiology, School of Public Health and Psychosocial Studies, Faculty of Health and Environmental Sciences, Auckland University of Technology, Auckland, New Zealand

##### **Correspondence:** Simon Otter

**Background**

Gout is the most common inflammatory arthritis in men and there has been a >60% increase in the prevalence of gout in recent years. Acute gout most frequently affects the foot - pain that does not fully resolve. This persistent nature of foot pain is reflected in ongoing lower-limb and foot-related impairment and disability. We aimed to determine the relationship between activity levels and foot function in people with gout.

**Methods**

Forty-nine people with gout (45 male, 4 female) were recruited from a rheumatology department in Auckland, New Zealand. All met the 1977 American Rheumatism Association classification criteria for gout (Trial registration: ACTRN12614001140640).

In addition to demographic variables, current medications, co-morbidities, gout disease duration, serum urate and presence of subcutaneous tophi were recorded for all participants.

Activity limitation was measured using the Health Assessment Questionnaire (HAQ). General body pain and first MTPJ pain over the past week were assessed using 100 mm VAS. Foot pain and disability was assessed using the Manchester Foot Pain and Disability Index. Lower limb disability during daily activities was assessed using the Lower Limb Task Questionnaire. Muscle strength for ankle plantarflexion, dorsiflexion, inversion and eversion was measured using a hand-held dynamometer. Foot type was assessed using the Foot Posture Index. Gait velocity was assessed using the six-meter walk test. Plantar pressure was assessed using a TekScan MatScan® system during barefoot walking at a self-selected speed. Peak plantar pressure (kPa) and pressure time integrals (kPa*s) were computed for seven masked regions of the plantar foot.

Differences in activity limitation by dichotomous variables were assessed using Mann-Whitney U tests and differences in activity limitation by ordinal variables were assessed using Kruskal-Wallis tests. Relationships were explored between activity limitation and continuous predictor variables using Spearman’s rho coefficients. Significant factors with a value of p < 0.15 were included in a stepwise multiple linear regression model and considered significant if p < 0.05.

**Results**

Participants had a mean age of 59 years (SD 13) and a mean gout disease duration of 17 years (SD 13). The mean HAQ score was 0.47 (SD 0.52).

Bivariate correlations were found between HAQ scores and gender, foot type, tophus count, generic pain, reduced daily and recreational activity, foot impairment, foot disability, reduced ankle plantarflexion, dorsiflexion, inversion and eversion muscle strength, increased pressure time integrals at the midfoot, lateral forefoot, lateral toes and reduced walking velocity.

With stepwise regression analysis, lower limb disability during daily activity, general body pain and increased pressure-time integral at the midfoot were independently associated with activity limitation (HAQ scores)

**Conclusions**

Reduced physical activity, general body pain and increased midfoot pressure-time integrals were predictors of impaired activity limitation. Significant correlation between impaired activity limitation and increased midfoot pressure time integral may reflect gait adaptation due to the prolonged disease duration. Our findings provide further insights into dynamic foot function in chronic gout and assists in the development of interventions for foot complaints in people with gout.

## 13 The effect of fatigue on the biomechanics of running

### Trevor Prior^1,2^, Andrea Bachand^3^, Ben Avison^3^, Jessica Leitch^3,4^

#### ^1^Centre for Sports and Exercise Medicine, William Harvey Research Institute, Bart’s and the London School of Medicine and Dentistry, Queen Mary University of London, London, UK; ^2^Homerton University Hospital NHS Foundation Trust, London, UK; ^3^Run3D Limited, The Bosworth Clinic, Oxford, UK; ^4^Department for Health, University of Bath, Bath, UK

##### **Correspondence:** Trevor Prior

**Background**

Fatigue decreases muscle force, decreases muscle control and causes biomechanical changes during running that can be detrimental to performance and increase susceptibility to injury [1, 2].

**Aim**

The aim of the study was to investigate the effect of fatigue on the biomechanics of running by comparing the kinematics of runners before and after a half-marathon (21 km) race. It was hypothesised that variables associated with injury and reduced muscular strength (Table 1) would be significantly different in the fatigued compared to the non-fatigued state.

**Methods**

Twelve male subjects (38.4 (8.3) years) attended two gait analysis sessions: one before and one immediately after completing a half-marathon. Reflective markers were placed on both lower-limbs and Vicon infrared cameras collected data at 200Hz as subjects ran on a treadmill. 3D joint angles were calculated and discrete parameters (Table 1) extracted from the data. Paired t-tests were used to compare the parameters before and after the half-marathon race.

**Results**

Cadence and knee-flexion angle significantly increased and stride-length and stance-time significantly decreased after the race compared to before (Table 1). No other statistically significant differences were found.

**Discussion**

Runners ran with increased cadence and knee flexion, and decreased stance-time and stride-length after racing a half-marathon. Although no other systemic alterations were found, further analysis revealed subject-specific changes suggesting that runners should be assessed individually in order to minimise the detrimental effects of fatigue on injury and performance.

**References**

1. Christina et al. Hum Movement Sci. **2001**,20,257

2. Miller et al. Gait Posture, **2007**,27,407.

**Table 1 (abstract 13):** See text for description

	BEFORE Mean(SD)	AFTER Mean(SD)
Hip-flexion foot-strike(deg)	26.7(4.5)	26.7(6.2)
Hip-extension toe-off(deg)	13.1(6.0)	11.4(7.8)
Peak hip-adduction(deg)	3.1(2.3)	-0.7(5.2)
Peak hip-rotation(deg)	16.3(7.2)	15.6(6.0)
Knee-flexion foot-strike(deg)*	13.2(4.9)*	16.2(6.4)*
Peak knee-rotation(deg)	14.4(5.1)	13.7(6.3)
Peak eversion(deg)	5.9(2.7)	5.0(3.1)
Stride-Rate(steps/sec)*	166(9.2)*	170.2(8.1)*
Stance-Time(sec)*	0.27(0.03)*	0.25 (0.03)*
Stride-Length(m)*	2.24(0.36)*	2.12(0.27)*

* significant difference

## 14 Efficacy of percutaneous flexor tenotomies for the management and prevention of recurrence of diabetic toe ulcers: A systematic review

### Jennifer Scott, Gordon Hendry, Jackie Locke

#### School of Health and Life Sciences Department, Glasgow Caledonian University, Glasgow, UK

##### **Correspondence:** Jennifer Scott

This abstract is not included here as it has already been published [1].

**Reference**

[1] Scott J, Hendry G, Locke J. Effectiveness of percutaneous flexor tenotomies for the management and prevention of recurrence of diabetic toe ulcers: a systemic review. J Foot Ankle Res 2016;9(25) http://doi.org/10.1186/s13047-016-0159-0

## 15 Can reducing the pH of wounds eliminate biofilm formation?

### Carla McArdle^1^, Katie Lagan^2^, David McDowell^2^

#### ^1^School of Health Sciences, Queen Margaret University, Edinburgh, UK; ^2^Faculty of Life and Health Sciences, Ulster University, Northern Ireland, UK

##### **Correspondence:** Carla McArdle

**Background**

Bacteria contaminate, colonize and often infect wounds of all types (Percival et al, 2015). Biofilms are composed of bacteria attached to each other and encased into an extracellular polymeric matrix, which very significantly increases their abilities to survive in otherwise hostile environments.

Within the last ten years, the presence and negative effects of biofilm in chronic wounds has been increasingly recognised with biofilm being reported as being a key virulence factor in the survival of bacteria in diverse milieu (Percival et al, 2012; Percival et al, 2014; Gilmore, 2015; Martin et al, 2015).

**Aim**

The aim of this study was to investigate if pH had any effect on biofilm formation.

**Methods**

This in vitro study used a representative sample of thirty wild type bacterial isolates recovered from diabetic wounds including, Pseudomonas species (5), Staphlococcus species including Methicillin Resistant Staphylococcus aureus (14), Escherichia. coli (4) and Streptococcus species (7). Tryptone Soya Broth (TSB) was prepared to a clinically relevant range of final pH values known to exist in wounds (pH 6.5 – 8.5) (Shukla et al, 2007; Shi et al, 2011; McArdle et al, 2015) using paired phosphate-diphosphate (Acros Organics, UK) and citric acid (Fisher Scientific, UK Ltd), buffers solutions, prepared and used as described by DeAngelis (2007) and Gomori (2010). Bacteria were added to the buffered TSB with final concentrations of ~102 cfu/ml calculated. Aliquots of inoculated (the presence of bacteria) buffered TSB and uninoculated buffered TSB (controls) were aseptically dispensed into rows (x8 replicate wells per row) in flat-bottomed 96 well plates and incubated aerobically for 24 h at 37 °C in the electronic plate reader (FLUOstar Omega, BMG labtech). The extent of biofilm production in each well was determined using the crystal violet binding assay as described by Stepanovic et al (2004) and an optical density reading obtained to quantify the biofilm formation.

**Results**

Both major groups of bacteria i.e. gram negative and gram positive are capable of producing moderate/strong biofilms at pH 8.5. In contrast to this, lowering the pH environment to that of less than pH 7 (acidic) has the ability to halt biofilm formation.

**Discussion and conclusion**

Wound status, in terms of healing of acute or chronic infection is determined by the balance between the ability of bacteria to establish and maintain infection within a wound environment and the ability of the host to control reduce or eliminate the bacterial community (Tadej et al, 2009). This study demonstrated that the range of pH values which have been shown to occur in wounds, can impact the biofilm production of bacterial communities. More specifically the majority of the isolates examined had the ability to produce more biofilm at alkaline pH values and less biofilm at acidic pH values. This exciting concept potentially means that selecting a dressing with an acidic pH may eliminate bacterial biofilm formation and thus the likelihood of persistent infection.

## 16 Factors associated with foot ulceration and amputation in adults on dialysis: A cross-sectional observational study

### Michelle Kaminski^1,2^, Anita Raspovic^1^, Lawrence McMahon^3^, Katrina Lambert^4^, Bircan Erbas^4^, Peter Mount^5^, Peter Kerr^6^, Karl Landorf^1,7^

#### ^1^Discipline of Podiatry, School of Allied Health, La Trobe University, Melbourne, Australia; ^2^Department of Podiatry, Eastern Health, Melbourne, Australia; ^3^Departments of Renal Medicine & Obstetric Medicine, Eastern Health Clinical School, Monash University, Melbourne, Australia; ^4^College of Science, Health and Engineering, School of Psychology and Public Health, Department of Public Health, La Trobe University, Melbourne, Australia; ^5^Department of Nephrology, Austin Health, Melbourne, Australia; ^6^Department of Nephrology, Monash Health, Melbourne, Australia; ^7^Melbourne Health, Melbourne, Australia

##### **Correspondence:** Michelle Kaminski

**Background**

Dialysis patients are at increased risk of foot ulceration, which commonly precedes more serious lower limb complications, including amputation. Limited data exist regarding the prevalence and factors associated with foot disease in this population. This study presents a cross-sectional analysis of baseline data from a multi-centre prospective cohort study, and investigates factors associated with foot ulceration and amputation in a dialysis cohort.

**Method**

450 adults with end-stage renal disease were recruited from satellite and home-therapy dialysis units in Melbourne, Australia between January and December 2014. Data collection consisted of a participant interview, medical record review, health-status questionnaire and non-invasive foot examination including neurological, arterial, biomechanical, footwear, and dermatological assessments. Logistic regression analyses were conducted to evaluate associations between screened variables and study outcomes.

**Results**

Mean age was 67.5 ± 13.2 years, 64.7% were male, 94% were on haemodialysis, median dialysis duration was 36.9 (IQR, 16.6 to 70.1) months, and 50.2% had diabetes. There was a high prevalence of previous ulceration (21.6%) and amputation (10.2%), 10% had current foot ulceration, and 50% had neuropathy and/or peripheral arterial disease. Factors associated with foot ulceration were previous amputation (OR, 10.19), peripheral arterial disease (OR, 6.16) and serum albumin (OR, 0.87); whereas previous and/or current ulceration (OR, 167.24 and 7.49, respectively) and foot deformity (OR, 15.28) were associated with amputation.

**Conclusions**

Dialysis patients have a high burden of lower limb complications. There are markedly higher risks of foot ulceration and/or amputation in those with previous and/or current ulceration, previous amputation, peripheral arterial disease, lower serum albumin, and foot deformity. Although not a major risk factor, diabetes in men was an important effect modifier for risk of ulceration. Early identification of those at greatest risk is essential for the prevention and management of foot complications and may improve outcomes. There is now a clear need for prospective studies to demonstrate if a temporal relationship exists between these factors and foot ulceration or amputation, and to evaluate interventions designed to reduce the risk of ulceration and amputation in the dialysis population.

## 17 An exploration of foot problems in people with Parkinson’s using foot and ankle assessments

### Louis Mamode^1^, Catherine Bowen^1^, Malcolm Burnett^1^, Lucy Gates^1^, Ann Ashburn^1^, Mark Cole^1^, Margaret Donovan-Hall^1^, Ruth Pickering^2^, Dan Bader^1^, Judy Robison^1^, Dorit Kunkel^1^

#### ^1^Faculty of Health Sciences, University of Southampton, Southampton, UK; ^2^Faculty of Medicine, University of Southampton, Southampton, UK

##### **Correspondence:** Louis Mamode

**Background**

Foot problems have been linked to instability and falls in the general population. People with Parkinson’s (PwP) are at particularly high risk of loss of balance than the general population but few researchers have explored foot problems among PwP. In this cross-sectional observational study (part of a larger project gathering information about indoor and outdoor footwear), we used an international musculoskeletal foot and ankle (IMFAA) protocol to explore foot problems in PwP.

**Methods**

Thirty PwP (survey respondents recruited from the larger study) mean age = 69, 15 females, mean time since diagnosis 64 months attended a single assessment at Southampton General Hospital. Those who were able to walk at least 5 metres, with or without a walking aid, were medically stable, able to answer simple questions and give informed consent were eligible for inclusion. Descriptive data on age, past medical history, time since diagnosis, more affected side, Hoehn and Yahr staging, Unified Parkinson’s disease Rating (UPDRS III) were recorded by a physiotherapist followed by foot status using an IMFAA protocol by a trained assessor (podiatrist). This measure includes 20 foot status measures including observation, palpation, Range of Motion, muscle strength and foot posture.

**Results**

The assessments revealed that all participants had foot problems (median 4, range 2-10) and of these, 12 (40%) currently received foot care support. The most common foot problems included: impairment or inability to perform a single limb heel raise (n = 21, 70%), hallux valgus (n = 19, 63%), impaired range of motion (n = 18 60%), foot pain (n = 17, 57%) or palpable abnormalities (n = 12, 40%). PwP fallers presented with a greater number of foot problems than non-fallers (p = 0.007). The number of foot problems correlated with lower balance confidence (r = 0.450, p = 0.013), lower balance scores on the MiniBESTest (r = -0.528, p = 0.003), slowed 5 m walking time (r = 0.528, p = 0.031) and with greater disease severity (Hoehn and Yahr stage r = 0.438, p = 0.015).

**Conclusions**

Using the IMFAA protocol in PwP revealed that foot problems are common in this group and may be associated with instability and falls. The question arises whether it might be possible to reduce the number of foot problems and decrease instability and risk of falls through early assessment and intervention of foot problems for PwP.

## 18 Prognostic factors for return to play following conservatively treated acute ankle sprain: A systematic review

### Saed Al Bimani^1,2,3,4^, Lucy Gates^1,3^, Martin Warner^1,2^, Catherine Bowen^1,2^

#### ^1^Faculty of Health Sciences, University of Southampton, Southampton, UK; ^2^Arthritis Research UK Centre for Sport, Exercise and Osteoarthritis, University of Southampton, Southampton, UK; ^3^Arthritis Research UK Centre for Sport, Exercise and Osteoarthritis, University of Oxford, Oxford, UK; ^4^Department of Physiotherapy, Institute of Health Sciences, Muscat, Oman

##### **Correspondence:** Saed Al Bimani

**Background**

Ankle sprain is a very common injury amongst the sport population yet uncertainty exists in what is appropriate time to return to play. Such guidance may inform treatment pathways and effective practice. The aim of this review is to determine if consensus exists about potential prognostic factors that are associated with return to play in conservatively treated acute ankle sprain.

**Method**

A systematic search of databases was conducted in DelphiS and EMBASE, unpublished literature and ongoing trials and two main ankle and foot injury sport-related journals. Inclusion criteria were full text studies in English assessing prognostic factors for return to sport following conservatively treated acute ankle sprain, any grade of injury of acute ankle sprain (new and recurrent), athletes practising any sport activity at whatever level, both male and female patients with age 14 and above. Four reviewers independently reviewed the quality of the included papers using Critical Appraisal Skills Programme criteria. One reviewer extracted relevant data.

**Results**

From review of literature, 2667 studies were identified by the initial search. Once the exclusion criteria were applied, a total of three studies were included in this review. One study recruited athletes from high schools whereas the other two studies recruited athletes who competed in the National Collegiate Athletic Association (NCAA). All studies recruited athletes with an age range of 14-22 years. Two studies used both subjective and objective prognostic factors and one study only used subjective prognostic factors. Return to play was not found to be associated with both new and recurrent injuries. Global function question, SF 36PF and athlete’s ambulation status, weight-bearing activity scores and self-reported athletic ability were stronger predictors for return to play. None of the included papers investigated participants’ demographic factors such as age, gender and BMI or history of injury as potential predictors of return to play. All studies used return to play as an outcome measure for recovery. Follow-up in all studies ranged from 1 to 40 days. All studies scored low level in the quality review.

**Conclusion**

This is the first review to report prognostic factors for return to play following conservatively treated acute ankle sprain. Consensus from three studies with methodological issues was not viable. Knowing prognostic factors will inform better practice related to evidence based decisions for return to play. Future prospective studies may consider recruiting bigger samples and investigating other demographic and history of injury as potential predictors for return to play.

## 19 Why do students come to study podiatry?

### Jane Murchie, Rachel Hannigan, Carla McArdle, Mairghread Ellis

#### School of Health Sciences, Queen Margaret University, Edinburgh, UK

##### **Correspondence:** Jane Murchie

**Background**

The shortage of healthcare workers has prompted research as to why individuals pursue a career in healthcare in order to improve recruitment and retention. This can particularly be seen in extensive research that has been carried out to examine what attracts individuals to a career in nursing. There is however a gap in the literature with regards to why individuals chose to study podiatry. An ageing population with complex co-morbidities places greater demand on podiatry services. It is therefore crucial that research is carried out to help focus recruitment strategies in order to target the right people to this essential profession. The rationale for undertaking this research was due to the current lack of evidence in this area and the need to investigate this further. Findings may also prove useful to the Society of Chiropodists and Podiatrists and other institutions on potential ways to promote the profession further in order to increase recruitment.

**Aim**

This study aims to investigate the underlying reasons as to why students come to study podiatry.

**Methods**

Students in years 1-3 of the Podiatry programme were recruited into this study. The Methodology used for this research was a mixed method approach via an online questionnaire (SurveryMonkey®). Questions allowed for open ended responses e.g ‘why did you chose to study podiatry?’ and questions which had a range of responses requiring the student to select what they deemed to be the most appropriate answer e.g ‘Did you know anything about Podiatry prior to starting the course’ and ‘how did you find out about podiatry?’. Thematic analysis was used to explore the qualitative data and descriptive statistics were calculated from the quantitative data.

**Results**

Forty participants took part in the online survey and thematic analysis of the qualitative responses revealed six main themes: previous experience of podiatry, choosing an alternative course prior to studying podiatry, an interesting profession, good career prospects, wanting to work in healthcare and a desire to help people. Quantitative data revealed that 69% of participants considered another course prior to selecting podiatry with the top three courses being physiotherapy, nursing and medicine. Forty six percent of participants found out about podiatry through family members or friends who are currently podiatrists or who have had experience of podiatry. School/careers advisors was one of the least used channels for participants to find out about the profession (17%).

**Discussion and Conclusion**

The general themes of helping people and wanting to work in health care were similar to research findings investigating why people study nursing (Eley et al. 2012; Wilkes et al. 2014). The lack of knowledge of podiatry within schools and careers offices indicates that higher education institutions need to gain an understanding of podiatry before they are able to direct potential students towards this career path. For future research it may be useful to investigate what influences other students across different universities to come to study podiatry, this would gain a greater insight into this research topic and provide potential ways to improve recruitment and retention strategies.

**Ethics statement:** Ethical permission was granted by Queen Margaret University, Edinburgh, divisional research ethics committee.

## 20 What do they really know about podiatry?

### Aimie Patience, Sophie Slater, Kirsten Wallace

#### School of Health Sciences, Queen Margaret University, Edinburgh, UK

##### **Correspondence:** Aimie Patience

**Background**

Previous research has identified a lack of understanding of podiatry when compared to their other AHP counterparts (Mandy et al, 2004). The confusion between the alternating terminology of chiropodist and podiatrist along with the historic ‘lone working’ of podiatrists is believed to contribute to the lack of understanding from the public and other health care workers (Vernon et al, 2005). Due to the importance of working within multidisciplinary teams with podiatry being an equal player in order to ensure high quality and holistic patient care, it was felt extremely important to establish if other AHPs were aware of the wide range of expertise that podiatrists have. This is an area which currently lacks research and therefore there a need for further studies to be conducted.

**Aim**

The research study aimed to investigate the understanding of podiatry among final year AHP students (physiotherapy, occupational therapy students etc) from a university in Scotland.

**Method**

Participants (N = 37) were recruited to identify their understanding of varying aspects of podiatric practice. The participants were asked to complete a survey consisting of 5 questions comprising of open-ended and closed questions, for example ‘in what NHS clinics/departments do you think podiatrists can work?’ The type of questions chosen intended to extract the participants understanding of podiatry while still allowing them to express their own opinions. Data was collected through the online survey (SurveyMonkey®) and the data was collated and analysed to identify common themes and draw conclusions.

**Results**

Eighty four percent of participants had heard of podiatry before beginning their AHP degree and 86% of participants knew podiatrists could remove toe nails. Seventy three percent did not know podiatrists could supply and administer certain medications with 46% not knowing that podiatrists could administer local anaesthetic, 86% however knew that podiatrists could perform minor surgical procedures for ingrowing toenails. Eighty four percent of participants stated they were aware that podiatrists worked in private clinics, for sports teams and in shoe shops. A number of participants commented on their awareness of podiatrists working in MSK clinics (32%), diabetes clinics (22%) and vascular units (14%). Furthermore 24% of participants stated that podiatrists could ‘supply or make’ insoles/orthotics and 46% mentioned that as well as looking after legs and feet, podiatrists could give health advice.

**Discussion and Conclusion**

This study showed that the majority of the participants had a good idea of a podiatrist’s role, with some areas being more widely acknowledged than others. One interesting observation of the findings was the number of participants who were unaware that podiatrists could administer LA but a vast majority knew that a podiatrist could perform nail surgery. While these results may differ from those of fully qualified AHPs, the overall findings however can be perceived as positive for the profession as it shows that future AHPs had a reasonably good overall understanding of the value of podiatry within the larger multidisciplinary team.

**Ethics statement:** Ethical permission was granted by Queen Margaret University, Edinburgh, divisional research ethics committee.

## 21 Evidence for current recommendations concerning the management of foot health for patients with chronic long-term conditions: A systematic review

### Katherine Edwards^1^, Alan M Borthwick^2^, Louise McCulloch^2^, Anthony C Redmond^3^, Rafael Pinedo-Villanueva^1,4^, Nigel K Arden^1,4,5^, Catherine J Bowen^2,5,6^

#### ^1^Nuffield Department of Orthopaedics, Rheumatology and Musculoskeletal Sciences, University of Oxford, Oxford, UK; ^2^Faculty of Health Sciences, University of Southampton, Southampton, UK; ^3^Faculty of Medicine and Health, Leeds Institute of Rheumatic and Musculoskeletal Medicine, Leeds, UK; ^4^MRC Lifecourse Epidemiological Unit, University of Southampton, Southampton, UK; ^5^Arthritis Research Centre for Sport, Exercise and Osteoarthritis, University of Oxford, Oxford, UK ^6^Arthritis Research Centre for Sport, Exercise and Osteoarthritis, University of Southampton, Southampton, UK

##### **Correspondence:** Katherine Edwards

**Background**

Evidence-based guidelines and standards of care are becoming increasingly popular, particularly in relation to diabetes and the diabetic foot. Consequently, UK NHS Podiatry services appear to have shifted their focus towards the management of acute wounds in the diabetic patient. Comparatively, guidelines for the management of foot health in patients with other chronic conditions are few. The aim of this investigation was to systematically review the evidence related to guidelines, standards of care and current recommendations concerning the management of foot health for patients with chronic long-term conditions.

**Methods**

Search criteria were defined by three experts in podiatry (CB, AR, and AB) through consensus agreement. The ‘PICO’ statement was used to establish inclusion criteria for the review. Electronic databases were searched for guidelines, standards of care and recommendations for the management of foot health in patients with chronic conditions published in English between 2000 and 2015. Additional references were identified from grey literature sources. The methodological quality of selected studies was assessed using the Appraisal of Guidelines for Research and Evaluation (AGREE II, 2010).

**Results**

The search strategy identified 5,352 citations, of which 153 met the review inclusion criteria. Of these, 90 (58.8%) were related to diabetes and the diabetic foot, 25 (16.3%) were on types of arthritis, 10 (6.6%) on peripheral arterial disease, six (3.9%) on other musculoskeletal conditions, and the remaining 22 (14.4%) were composed of small numbers of references relating to other conditions. Methodological assessment showed that 42 (27.5%) of included papers met the AGREE II criteria for level 7 evidence, which comprised predominantly of diabetes-related medically-led guidelines and standards of care (e.g. NICE, SIGN). More than half (81, 52.9%) of included papers were classified as ‘expert opinion and good clinical practice’, i.e. level 4 quality evidence or lower.

**Conclusions**

This review identified a number of published guidelines, standards of care and recommendations that included foot health in patients with chronic conditions. Findings show that the literature is predominantly comprised of those relating to the treatment and management of diabetes and the diabetic foot. The high quality guidelines and standards of care that exist are primarily those that are medically driven with the main focus on medical and pharmacological management with only indirect reference to general foot care. There is a distinct lack of high quality evidence in the literature to guide recommendations for foot care in patients with other chronic conditions, particularly general podiatry and foot pain.

Prospero Registration: CRD42015020278.

## 22 Points to consider for health professionals undertaking musculoskeletal ultrasound for rheumatic and musculoskeletal diseases: Progress of a EULAR Task Force

### Heidi Siddle^1^, Peter Mandl^2^, Daniel Aletaha^2^, Thea Vliet Vlieland^3^, Marina Backhaus^4^, Patricia Cornell^5^, Maria Antonietta D'Agostino^1,6^, Karen Ellegaard^7^, Annamaria Iagnocco^8^, Bente Jakobsen^9^, Tiina Jasinski^10^, Nina Kildal^11^, Michaela Lehner^2^, Ingrid Moller^12^, Gabriela Supp^2^, Philip O’Connor^13^, Anthony Redmond^1^, Esperanza Naredo^14^, Richard Wakefield^1^

#### ^1^Leeds Institute of Rheumatic and Musculoskeletal Medicine, University of Leeds, Leeds, UK; ^2^Division of Rheumatology, Medical University of Vienna, Vienna, Austria; ^3^Department of Orthopaedics, Rehabilitation and Physical Therapy, Leiden University Medical Centre, Leiden, Netherlands; ^4^Department of Internal Medicine, Rheumatology and Clinical Immunology, Academic Hospital of the Charité Berlin, Berlin, Germany; ^5^Department of Rheumatology, Poole Hospital NHS Foundation Trust, Poole, UK; ^6^Laboratoire d’Excellence INFLAMEX, Versailles-Saint-Quentin University, Saint-Quentin-en-Yvelines, France; ^7^The Parker Institute, Copenhagen University Hospital Bispebjerg-Frederiksberg, Copenhagen, Denmark; ^8^Rheumatology Unit - Dipartimento di Medicina Interna e Specialità Mediche, Sapienza Università di Roma, Rome, Italy; ^9^Norwegian National Advisory Unit on Pregnancy and Rheumatic Diseases, St. Olavs Hospital, Trondheim, Norway; ^10^Estonian Rheumatism Association, Tallinn, Estonia; ^11^Faculty of Health and Social Science, Norwegian University of Science and Technology, Trondheim, Norway; ^12^Instituto Poal de Reumatologia, Barcelona, Spain; ^13^Department of Radiology, Leeds Teaching Hospitals NHS Trust, Leeds, UK; ^14^Department of Rheumatology, Hospital GU Gregorio Marañón, Madrid, Spain

**Background**

Musculoskeletal ultrasound (MSUS) has evolved into an important method for identifying musculoskeletal abnormalities, confirming the diagnosis in patients with suspected inflammatory arthritis, monitoring therapeutic response, influencing clinical decision making and guiding interventions. The role of the non-medical health professional has advanced with many health professionals undertaking training and using MSUS to extend their scope of practice. Guidelines to support training for rheumatologists have been identified but currently there are not any recommendations to support the education and training needs of non-medical health professionals using MSUS.

**Objectives**

A European League Against Rheumatism (EULAR) task force was established to reach a consensus on the role and education and training needs of non-medical health professionals undertaking MSUS for the management of patients with rheumatic and musculoskeletal diseases (RMDs).

**Methods**

The EULAR Task Force comprised an expert group from 10 European countries. The Task Force met twice and included rheumatologists, nurses, physiotherapists, a radiologist, radiographer and podiatrist with patient representation. At the first meeting the aims of the Task Force were defined and fourteen research questions were developed. This was followed by a comprehensive systematic search of the literature to identify publications on the role of health professionals using MSUS, their competency requirements and training undertaken. During the second meeting the Task Force were presented with the results of the literature review which supported the formulation and consensus of ‘points to consider’. The level of agreement between task force members for each ‘point to consider’ was determined after the second meeting.

**Results**

In total, seven ‘points to consider’ were formulated. Two points covered the role and scope of health professionals using musculoskeletal ultrasound and one point addressed the application and feasibility in daily practice. Three ‘points to consider’ focused on the training and competency required by health professionals and the final point addressed the added value in daily practice. The strength of the ‘points to consider’ was D based on the category of evidence (3-4). A high level of agreement (range 9.0-9.7) was achieved between Task Force members. Additionally, the Task Force agreed upon eight topics for the future research agenda.

**Conclusion**

Seven ‘points to consider’ for health professionals using MSUS for the management of patients with RMDs were developed using a combination of research-based evidence and expert consensus approach. The seven points to consider are intended to support the education and training needs for non-medical health professionals using MSUS to improve the management of patients with RMDs across Europe, in conjunction with local and national regulations.

